# Optimizing Lenvatinib Therapy for Prognostic Improvement in Advanced Thyroid Cancer

**DOI:** 10.3390/ph18101432

**Published:** 2025-09-24

**Authors:** Tetsuro Wakasugi

**Affiliations:** Department of Otorhinolaryngology-Head and Neck Surgery, University of Occupational and Environmental Health, 1-1 Iseigaoka Yahatanishi-ku, Kitakyushu 807-8555, Fukuoka, Japan; wakasugi@med.uoeh-u.ac.jp; Tel.: +81-93-691-7448

**Keywords:** lenvatinib, radioiodine-refractory differentiated thyroid cancer, planned drug holiday, relative dose intensity, neoadjuvant therapy, conversion surgery, dose re-escalation, rechallenge

## Abstract

Radioiodine-refractory differentiated thyroid cancer (RAIR-DTC) is associated with poor prognosis and limited systemic options. This narrative review focuses on lenvatinib (LEN), a multitarget tyrosine kinase inhibitor that significantly prolongs progression-free survival. Evidence from the SELECT trial and real-world data indicates that its benefits can be enhanced through early initiation, maintenance of a high relative dose intensity, and proactive toxicity management. Planned drug holidays help sustain treatment while avoiding prolonged unplanned interruptions. In selected patients with locally advanced, initially unresectable disease, neoadjuvant LEN may enable conversion surgery, facilitating subsequent treatments. However, the current data are mainly from case series and early-phase studies. After dose reduction, re-escalation can restore disease control, and LEN rechallenge after a drug-free interval may restore sensitivity in later lines. Thus, LEN should be integrated into personalized multidisciplinary care to optimize outcomes across treatment courses. Nevertheless, key limitations remain, as much of the supporting evidence is derived from post hoc or retrospective analyses. Prospective studies are required to validate the optimization strategies, define stage-specific benefits, and determine their impact on overall survival.

## 1. Introduction

Radioiodine-refractory differentiated thyroid cancer (RAIR-DTC) has long been associated with a poor prognosis owing to the lack of effective systemic therapies after radioactive iodine (RAI) treatment failure [1]. This review synthesizes and critically evaluates current evidence and emerging strategies aimed at optimizing lenvatinib (LEN) therapy in RAIR-DTC. The therapeutic landscape began to shift with the DECISION trial, which demonstrated that sorafenib modestly prolonged progression-free survival (PFS; median 10.8 vs. 5.8 months) but yielded a low objective response rate (ORR; 12.2%) [2]. These limitations underscored the need for more effective therapies, paving the way for LEN. LEN is a multitarget tyrosine kinase inhibitor (MKI) that inhibits VEGFR1–3, FGFR1–4, RET, KIT, and PDGFRα. In the pivotal SELECT trial, LEN demonstrated significantly improved PFS (median 18.3 vs. 3.6 months; HR 0.21, 99% CI 0.14–0.31) and an ORR of 64.8% compared to placebo (PBO), thereby establishing itself as the current standard of care for RAIR-DTC [3]. However, further optimization is required to maximize the clinical benefits of LEN. Accumulating post hoc analyses of the SELECT study and real-world evidence suggest that outcomes can be further improved by optimizing several aspects of LEN therapy, including appropriate patient selection, early treatment initiation, maintenance of high-dose intensity in the initial treatment phase, and proactive management of adverse events (AEs). These findings, while promising, remain exploratory and require prospective validation. Furthermore, in prospective trials conducted after the SELECT trial, planned drug holidays have emerged as a strategy for managing toxicity without compromising efficacy, allowing longer treatment durations and quality of life improvements.

In addition to these strategies, novel applications of LEN have also gained attention. Notably, LEN shows promise as a neoadjuvant therapy for patients with locally advanced and initially unresectable thyroid cancer, facilitating conversion surgery and curative resection. Furthermore, new therapeutic approaches, such as re-escalation in the first-line setting and the re-administration of third-line treatment, have demonstrated the potential for additional therapeutic effects of LEN.

This review aimed to comprehensively summarize the current evidence regarding LEN therapy for RAIR-DTC by focusing on strategies to maximize its therapeutic potential. As the therapeutic paradigm shifts toward more personalized and adaptive care models, understanding the full spectrum of LEN’s clinical utility is essential for improving long-term outcomes in patients with advanced thyroid cancer. By enabling durable disease control, LEN not only improves short-term outcomes but may also contribute to long-term survival, underscoring its pivotal role in shaping the evolving treatment paradigm of RAIR-DTC. Contextualizing LEN within the broader therapeutic landscape, while addressing both established evidence and remaining challenges, provides a framework for integrating LEN into more personalized, multidisciplinary strategies. Ultimately, LEN should be regarded not only as a potent systemic agent but also as a cornerstone therapy with the potential to improve long-term survival in patients with RAIR-DTC.

## 2. Strategies to Maximize Therapeutic Benefit of First-Line LEN Therapy

### 2.1. Mechanism of Action of LEN

Differentiated thyroid cancer (DTC) is characterized by frequent alterations in the signaling pathways that drive tumor growth and survival. Mutations in the MAPK pathway—most notably BRAFV600E and RAS—lead to the constitutive activation of downstream signaling and enhanced tumor cell proliferation and survival. Alterations in the PI3K/AKT pathway, including PTEN loss, contribute to oncogenic signaling and tumor progression [4].

A hallmark of thyroid carcinogenesis is the overexpression of vascular endothelial growth factor (VEGF) and its receptors (VEGFRs), which are strongly associated with tumor angiogenesis and poor prognosis [4]. Based on these findings, various VEGFR-targeted tyrosine kinase inhibitors (TKIs) were first evaluated in phase II trials and subsequently advanced to phase III studies. Sorafenib (DECISION trial) and LEN (SELECT trial) were the first agents to demonstrate significant clinical benefits in phase III settings, thereby establishing VEGFR inhibition as a validated therapeutic approach.

LEN is a potent multi-target TKI that inhibits VEGFR1–3, FGFR1–4, PDGFRα, RET, and KIT [5]. Notably, LEN is unique among TKIs in its ability to target FGFRs, which are overexpressed in thyroid cancers and implicated in the mechanism of resistance to VEGF-targeted therapy. FGFR upregulation has been described as a key evasive pathway that enables tumors to escape blockade; thus, the ability to inhibit FGFR1-4 offers a unique advantage in overcoming such resistance. Its high affinity for VEGFR2–3 further augments its antiangiogenic potency. By simultaneously inhibiting VEGFR, FGFR, PDGFRα, RET, and KIT, LEN effectively blocks tumor angiogenesis and tumor progression, leading to potent antitumor effects. In contrast, sorafenib exerts relatively weaker VEGFR inhibition, but also targets c-KIT and BRAF [6].

In addition to these direct antiangiogenic and anti-proliferative effects, emerging preclinical evidence suggests that LEN can modulate the tumor immune microenvironment. This immunomodulatory potential creates a rationale for its combination with immune checkpoint inhibitors (ICIs). These immunological effects and their clinical implications are discussed in Section 3.3.

### 2.2. Clinical Evidence from SELECT, Real-World Data, and Comparisons

As previously noted, in the pivotal SELECT trial, LEN demonstrated significantly improved PFS compared with PBO in patients with RAIR-DTC, achieving a median PFS of 18.3 months versus 3.6 months (hazard ratio [HR], 0.21; 99% confidence interval [CI], 0.14–0.31) and an ORR of 64.8% [2]. Real-world clinical data on the first-line treatment of LEN with RAIR-DTC in the U.S. were subsequently published, suggesting that the clinical benefit in practice was even greater than the SELECT results. In this report, the median PFS reached 49 months, and the best overall response rate increased to 72.4% [7]. These differences may reflect advances in supportive care, higher treatment continuity, and distinct baseline patient characteristics, underscoring LEN’s potential for enhanced efficacy in certain patient populations. Accordingly, the current National Comprehensive Cancer Network (NCCN) guidelines for thyroid carcinoma list both LEN and sorafenib as category 1 systemic therapies for progressive RAIR-DTC [8]. However, LEN is designated as the preferred first-line option based on its substantially higher ORR and greater depth and durability of response demonstrated in the SELECT trial and supported by real-world evidence showing longer PFS and improved treatment continuity.

Comparison with other systemic therapies further underscores the superior efficacy of LEN. In the DECISION trial, sorafenib achieved only modest benefit with a median PFS of 10.8 versus 5.8 months and an ORR of 12.2%. Cabozantinib, as evaluated in the COSMIC-311 trial, provided clinical benefit in the post-LEN setting (median PFS 11.0 months; ORR 11.0%) but remained less effective than LEN as an initial therapy [9].

Immunotherapy monotherapy has not yet been established in RAIR-DTC. However, early exploratory studies are underway, and novel combination approaches are actively being investigated (see Section 3.3).

Collectively, these data confirm LEN as the cornerstone of first-line systemic therapy for RAIR-DTC. Sorafenib and cabozantinib are positioned in subsequent lines, while immunotherapy remains investigational. Table 1 summarizes the major efficacy outcomes from pivotal trials. As shown, LEN demonstrated superior first-line efficacy compared to sorafenib, whereas cabozantinib had established benefits in the post-LEN setting. Immunotherapy remains investigational, although emerging data suggests a potential synergy in combination with LEN. Therefore, LEN should be prioritized as the standard first-line treatment option for RAIR-DTC.

### 2.3. Timing of Treatment Initiation

In the SELECT trial, no statistically significant difference in overall survival (OS) was observed between the LEN and PBO groups (HR, 0.73; 95% CI, 0.50–1.07; *p* = 0.1032) [3]. This result is largely attributable to the trial design, which allowed crossover from PBO to LEN upon progressive disease (PD). Notably, 83% of the patients in the PBO arm initiated LEN treatment after PD confirmation. The rank-preserving structural failure time model was applied to address the confounding effects of crossover. This exploratory analysis revealed a significant OS benefit in the LEN arm (HR, 0.53; 95% CI, 0.34–0.82; *p* = 0.0051), suggesting that a delayed initiation of LEN may attenuate its therapeutic impact [11]. These findings suggest that the timing of LEN initiation plays a pivotal role in optimizing clinical outcomes. In the SELECT study population, in which crossover was allowed, there was a mix of early- and late-start cases. Therefore, the variables listed in Table 2 that showed significant differences between the LEN and PBO groups may partly reflect the consequences of delayed treatment rather than the intrinsic characteristics of the patient or disease [12,13,14,15]. From this perspective, patients exhibiting these specific factors may be particularly suitable for early LEN initiation. It should be noted, however, that these high-risk features were identified through subgroup and post hoc analyses and thus remain exploratory. Prospective validation is needed before it can be incorporated into routine clinical decision making.

Further analysis of the LEN treatment arm of the SELECT trial provided new insights into prognosis. As summarized in Table 3, several clinical and demographic factors were associated with differences in OS in the LEN group [15,16]. This indicates that patients with unfavorable prognostic factors have a poor prognosis despite receiving LEN. Importantly, these factors may reflect a delay in treatment initiation; in other words, the potential efficacy of LEN may be reduced when administered after PD or functional decline.

Timely therapeutic intervention in such cases may maximize clinical benefits, highlighting the importance of strategic treatment timing in the management of RAIR-DTC. Therefore, the presence of such factors at the start of LEN treatment may be an indicator of optimal early treatment initiation. Initiating LEN before the development of adverse pathologies may improve survival outcomes. These observations reinforce the potential utility of early intervention in the selection of patients with RAIR-DTC and warrant further investigation in prospective studies.

The NCCN Clinical Practice Guidelines state that systemic therapy should be considered for “progressive and/or symptomatic disease” [8]. However, data from these sub-analyses of the SELECT trial suggest that certain patient characteristics may influence prognosis depending on the initiation of LEN. In particular, factors that showed significant differences between the LEN and PBO groups (age >65 years, lung metastases >1 cm, follicular cancer, and baseline tumor diameter >40 mm) may be indicators of the survival benefits of early treatment initiation. These variables are likely to reflect the impact of treatment delay, supporting the hypothesis that early intervention optimizes clinical outcomes. Moreover, prognostic factors such as a baseline performance status (PS) of 1, neutrophil-to-lymphocyte ratio (NLR) of ≥3, and baseline tumor volume of ≥40 mm were associated with OS among the LEN-treated cohort. As these factors tend to worsen over time and are associated with a poor prognosis if a patient is already deteriorating, there may be an advantage to starting LEN before such deterioration occurs, that is, early use. Taken together, these findings advocate a more nuanced and risk-adapted approach to the timing of systemic therapy in RAIR-DTC beyond the traditional “progressive or symptomatic” criteria. Thus, the early initiation of LEN should be considered in patients presenting with high-risk features predicted to have a poor prognosis if treatment is delayed.

### 2.4. Optimization of Dosing Strategies

The optimization of dosing strategies is essential to maximize the clinical efficacy of LEN in RAIR-DTC. In the SELECT trial, the starting dose of LEN (24 mg once daily) significantly improved PFS and ORR in patients with RAIR-DTC versus PBO. However, owing to the frequent treatment-emergent AEs (TEAEs) at this dose, attempts have been made to reduce the starting dose in clinical practice. Therefore, a randomized controlled trial was conducted to investigate whether a reduction in the starting dose to 18 mg would provide equivalent efficacy while improving tolerability [10]. In this trial, the ORR at week 24 was significantly higher in the 24 mg group (57.3%) versus 18 mg group (40.3%), with an odds ratio of 0.50 (95% CI, 0.26–0.96); non-inferiority of the 18 mg dose was not demonstrated. Notably, the incidence of grade ≥3 TEAEs at week 24 was similar between groups (24 mg, 61.3% vs. 18 mg, 57.1%).

This suggests that a reduction in the starting dose does not significantly improve safety and that a starting dose of 24 mg is justified by its superior efficacy. Importantly, post hoc analyses of the SELECT trial revealed that patients who achieved an objective response—complete or partial—had a significantly longer PFS (HR [95% CI], 0.61 [0.41–0.91]; *p* = 0.014) compared to those with stable disease or PD [17]. Furthermore, early tumor shrinkage (at 8 weeks) was significantly correlated with higher LEN exposure (area under the curve), underscoring the importance of maintaining adequate LEN exposure during the initial 8-week period. As the early treatment phase carries the highest risk of adverse events, blood pressure and proteinuria should be monitored weekly during weeks 1–8 to enable early detection and timely intervention. This practical approach supports the safe maintenance of dose intensity. Taken together, these findings emphasize the clinical importance of starting treatment at 24 mg, maintaining adequate LEN exposure in the initial phase during the first 8 weeks after initiation to maximize its therapeutic efficacy.

### 2.5. Limitations in Specific Patient Subgroups

Although LEN has demonstrated substantial efficacy in many patients with RAIR-DTC, its prognostic benefit may be limited and, in some cases, potentially harmful in specific patient populations. For example, tumors that directly invade critical anatomical structures, such as the skin, trachea, major blood vessels, or esophagus, pose a considerable risk of life-threatening complications, such as fistula formation or catastrophic hemorrhage. In such cases, LEN-induced tumor shrinkage may precipitate fatal events [18,19]. In the SELECT trial, fatal AEs were reported in 7.7% of patients receiving LEN, and 2.3% of patients experienced treatment-related deaths, including pulmonary embolism, hemorrhagic stroke, and sudden death [3].

Additionally, patients with significant hepatic or renal dysfunction may be unable to tolerate high-dose LEN, thereby limiting the feasibility of early intensive dosing strategies associated with improved outcomes. For these individuals, a dose-intensification approach aimed at maximizing the tumor response may be impractical.

Furthermore, the role of LEN is limited in patients harboring specific genetic alterations, such as *BRAFV600E*, *RET*, or *NTR*K fusions, particularly in anaplastic thyroid carcinoma or medullary thyroid carcinoma, where the role of LEN is limited. In these settings, molecularly targeted therapies are available and are recommended as preferred options in the current NCCN guidelines [8]. In DTC, these targeted agents have demonstrated efficacy in patients with corresponding alterations. However, no head-to-head comparisons with LEN have been conducted; thus, the appropriate use of LEN remains a reasonable first-line option in such cases.

With the expanding armamentarium of systemic therapies, including selective kinase inhibitors and immune-based treatments, careful molecular and anatomical assessments are essential to determining the appropriateness of LEN. Alternative treatment strategies may provide superior survival benefits in these subgroups; nevertheless, LEN remains the rational first-line choice for appropriately selected patients.

### 2.6. Adverse Event Management

Effective management of treatment-emergent adverse events (TEAEs) during long-term treatment is essential for LEN to contribute meaningfully to the improved survival of patients with RAIR-DTC. A post hoc analysis of the SELECT trial reported that patients who developed grade ≥2 hypertension experienced better prognoses, suggesting that certain TEAEs may reflect adequate drug exposure and therapeutic activity [20]. In the SELECT study, any-grade AEs occurred in 97.3% of the patients. The most frequent grade ≥3 AEs were hypertension (44%), proteinuria (31%), weight loss (10%), diarrhea (8%), and decreased appetite (5%) [3]. The most common AEs of any grade (≥10%) included hypertension (73%), diarrhea (67%), decreased appetite (50%), weight loss (46%), hand–foot syndrome (32%), thyroid-stimulating hormone elevations (30%), and stomatitis (24%). Management should be tailored to the severity of the AEs. Grade 1–2 events can generally be managed with prompt supportive care (e.g., antihypertensives, emollients, and antidiarrheal agents) and continued therapy. For AEs such as proteinuria or decreased appetite, where supportive measures may be limited, temporary interruption is recommended until improvement. For grade ≥3 events, treatment interruption is essential, and dose reduction is advised when therapy is resumed after recovery. Notably, retrospective real-world data indicate that even in patients who develop grade ≥3 proteinuria, irreversible declines in estimated glomerular filtration rate (eGFR), dialysis requirement, or permanent treatment discontinuation are uncommon, and renal function often improves after drug interruption. Diabetes mellitus was identified as an independent risk factor for greater eGFR decline, underscoring the importance of vigilant renal monitoring. These findings support the concept that temporary interruption with subsequent dose adjustment allows the safe continuation of LEN rather than necessitating permanent cessation, even in cases of higher-grade proteinuria [21].

In clinical practice, maintenance dosing after the initial 24 mg dose is individualized according to tolerability and AE management. Stepwise reductions—typically to 20, 14, and 10 mg daily, with further reductions to 8 or 4 mg if needed—are guided by tolerability and AE management. Dose management combined with close monitoring of blood pressure and proteinuria monitoring weekly during the first month, every two weeks thereafter, and monthly once stable, together with patient self-monitoring, enables early detection and timely management. [22]. Timely and proactive dose modifications are critical to balance efficacy and tolerability, thereby maintaining an adequate RDI and supporting long-term efficacy.

A multidisciplinary approach involving physicians, pharmacists, and nurses facilitates early detection, patient education, and rapid intervention, all of which are key to sustaining treatment and maximizing clinical benefits. Real-world studies and expert guidance emphasize the importance of structured outpatient systems that allow rapid dose adjustment and coordinated supportive care without compromising efficacy [22,23,24]. Such proactive, team-based management not only preserves therapeutic exposure and RDI but may also help prevent hospitalization and reduce overall healthcare costs. Planned drug holidays are particularly important. Prospective evidence, optimal scheduling, and the impact on long-term outcomes.

### 2.7. Planned Drug Holidays

**Figure 1:**
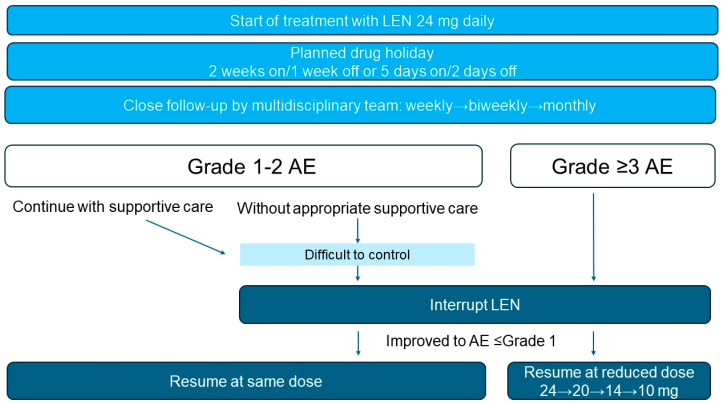
Management Algorithm for Lenvatinib Therapy with Planned Drug Holidays and AE–Based Dose Modification.

In the SELECT trial, the prolonged interruption of LEN treatment was associated with significantly worse PFS. The key concepts and management algorithms described in Section 2.6 and Section 2.7 have been integrated and are presented collectively in Figure 1. Patients with <10% treatment interruptions experienced a median PFS that was not estimable (NE; 95% CI, NE–NE), whereas those with ≥10% treatment interruptions had a substantially shorter median PFS of 12.8 months (95% CI, 9.3–16.5) [25]. Notably, treatment interruptions occurred in 82.4% of patients in the SELECT trial [3], indicating that temporary discontinuation of treatment is virtually inevitable in real-world clinical practice.

Scheduled withdrawals have been explored as a strategy to avoid prolonged unplanned treatment interruptions. Retrospective studies have also reported better treatment outcomes in patients undergoing planned drug holidays [26]. Based on these findings, the prospective COLLECT study conducted in Japan evaluated the impact of planned withdrawal on clinical outcomes. In this trial, LEN was initiated at 24 mg daily, with a dose reduction permitted in case of AEs. Planned drug holidays were also allowed to prevent severe or intolerable toxicities, and 28.9% of the patients underwent such holidays at the discretion of the treating physician. Patients demonstrated significantly better outcomes in the planned versus unplanned withdrawal group. The HR values strongly favored planned withdrawal of PFS (HR, 0.30), OS (HR, 0.311), time to treatment failure (HR, 0.42), and time to subsequent therapy (HR, 0.449) [27]. All endpoints were significantly longer than those in the non-holiday group, with a particularly favorable 1-year OS (95.8%) and 1-year PFS (94.5%) in the planned holiday cohort. Furthermore, the duration of maintaining a dose ≥10 mg was significantly longer in the planned withdrawal group.

Interestingly, the planned-holiday cohort showed an overall higher incidence of Grade ≥3 adverse events, which likely reflects the longer cumulative duration of maintaining doses ≥10 mg. Importantly, however, no cases of Grade ≥3 fistula formation occurred in the planned-holiday group, whereas such events were observed in 2.8% of the non-holiday group [27]. This suggests that proactively scheduled drug holidays may help prevent the most serious adverse events, thereby avoiding hospitalization, which, in turn, has the potential to reduce overall healthcare costs.

Furthermore, a retrospective study showed that maintaining an RDI above 60% during the first 8 weeks was significantly associated with improvements in PFS and OS [28]. Based on this finding, we calculated the extent of scheduled withdrawal that would maintain an 8-week RDI (8w-RDI) of at least 60%. For a treatment regimen starting at 24 mg daily, a “2 weeks on/1 week off” schedule would yield an 8w-RDI of 66.7% (average daily dose of 16.0 mg), while a “5 days on/2 days off” weekly cycle would result in an 8w-RDI of 71.4% (average daily dose, 17.1 mg).

These planned interruptions can preserve adequate dose intensity. However, in real-world clinical practice, reduced starting doses are sometimes necessary, particularly in frail or older patients. Such lower starting doses are unlikely to meet the 8w-RDI ≥60% threshold and may therefore not fully deliver the therapeutic benefit of LEN.

This highlights the importance of individualized treatment planning that balances toxicity management with the need to maintain a sufficient initial dose intensity to optimize outcomes in patients with RAIR-DTC. Furthermore, findings from the COLLECT study and other real-world analyses indicated that scheduled drug holidays can be proactively implemented without compromising therapeutic efficacy, provided that adequate RDI is maintained. Incorporating structured breaks as part of a proactive treatment plan may help reduce toxicity while preserving antitumor activity and ultimately improving patient outcomes.

### 2.8. Dose Adjustment and Rechallenge

LEN is well established as a first-line therapy for RAIR-DTC, but its role can extend beyond initial treatment through two key strategies: (1) dose re-escalation following prior reduction; and (2) rechallenge in later lines of therapy. Both approaches aim to sustain disease control, delay PD, and potentially improve the survival of appropriately selected patients.

Dose re-escalation is particularly indicated for patients whose prior dose reductions in LEN were undertaken to mitigate TRAEs and who subsequently experienced tumor progression when AEs were stable and re-escalation was clinically feasible. In patients who have undergone dose reduction to manage AEs during long-term therapy, PD may indicate that the current LEN dose is insufficient to control tumor growth. The observation that dose re-escalation is effective may, at least in part, be explained by this rationale.

In such cases, if toxicities are manageable, re-escalating the dose to restore drug exposure to a level commensurate with the tumor burden may enable the recapture of disease control. These findings underscore the importance of early proactive AE management to facilitate successful re-escalation. Importantly, the appropriateness of re-escalation presupposes that AE management is already well-established, and careful toxicity monitoring remains particularly critical during and after dose re-escalation to ensure patient safety.

In a retrospective study of 33 patients [29], dose re-escalation by a median of 6 mg from the reduced dose resulted in a median OS of 20.5 months compared with 3.9 months for those who discontinued therapy (*p* = 0.0004; HR, 0.22). The ORR was 13.3%, clinical benefit rate was 73.3%, and median treatment duration was 9.9 months, with no notable increase in severe AEs following re-escalation.

Following LEN failure in RAIR-DTC, second-line ORRs are reportedly 15.5% for cabozantinib and 11.1% for sorafenib [30]. Although these data originated from separate studies and direct cross-trial comparisons are limited, the magnitude of the response is broadly comparable to that observed with LEN re-escalation. This suggests that in appropriately selected patients, LEN re-escalation may be considered before transitioning to an alternative targeted agent, thereby prolonging disease control with a previously effective therapy, deferring the need for drugs with different toxicity profiles, and potentially optimizing treatment sequencing strategies in RAIR-DTC.

Rechallenge of LEN has emerged as a promising strategy even in later lines of therapy. In a cohort study by Yokota et al., among 95 patients with RAIR-DTC treated with first-line LEN (median PFS, 20.4 months), 41 received second-line sorafenib and 16 were subsequently rechallenged with LEN as third-line therapy. The rechallenge achieved a disease control rate of 100% and a median PFS of 15.0 months with no new safety concerns. A median LEN-free interval of 5.7 months may have contributed to the restoration of drug sensitivity, supporting the feasibility of LEN rechallenge in patients with limited treatment options [31].

At present, beyond LEN refractoriness, second-line options include sorafenib and cabozantinib; however, there are no established and effective third-line standard therapies for RAIR-DTC, leaving patients with limited treatment options. In this context, both dose re-escalation and rechallenge represent practical and effective strategies for extending the clinical utility of LEN beyond first-line treatment. For patients without access to alternative targeted agents or clinical trials, these approaches may offer continued disease control and improve long-term outcomes.

LEN is an MKI that inhibits VEGFR, FGFR, PDGFRα, KIT, and RET, among others, and the mechanisms of resistance remain incompletely understood. For example, upregulation of the EGFR/ERK/AKT pathway has been observed in thyroid cancer cells with acquired LEN resistance, while co-administration of the EGFR inhibitor lapatinib has been shown to restore LEN sensitivity [32]. In addition, epithelial–mesenchymal transition, alterations in ferroptosis, and RNA modifications—factors linked to the tumor microenvironment and epigenetic regulation—have been implicated in the development of resistance. These mechanistic insights provide a plausible biological rationale that supports the retrospective evidence of LEN rechallenge efficacy [33].

These findings demonstrate that LEN re-escalation and rechallenge are not only feasible but may provide clinical benefits comparable to, or even exceeding, existing second-line therapies in selected patients. Mechanistic evidence of restored sensitivity after a LEN-free interval further strengthens the rationale for rechallenge as a valid later-line strategy.

Across the treatment continuum for RAIR-DTC, LEN’s utility can be maximized through: (1) proactive toxicity management via planned drug holidays to sustain dose intensity; (2) preoperative use to enable curative conversion surgery in locally advanced disease; and (3) re-escalation or rechallenge to extend the benefits beyond first-line therapy. Together, these strategies position LEN as a potent MKI as well as a versatile, adaptable backbone of personalized, multidisciplinary thyroid cancer care.

## 3. Expanding Clinical Utility and Future Perspectives

### 3.1. Emerging Biomarkers

In addition to established clinical prognostic factors, several exploratory biomarkers have been investigated as potential predictors of LEN efficacy in RAIR-DTC. Eastern Cooperative Oncology Group performance status (ECOG PS) and the neutrophil-to-lymphocyte ratio (NLR) have already been identified as significant prognostic factors in post hoc analyses of the SELECT trial [12,16]. Additional exploratory markers have since been reported. Nutritional status assessed using the Controlling Nutritional Status (CONUT) score has shown correlation with prognosis because it reflects host conditions and systemic inflammation [34]. Moreover, ^18^F-FDG PET/CT has emerged as a promising dynamic imaging biomarker, and early metabolic changes, including SUVmax reduction within the first weeks of LEN therapy, were associated with improved subsequent PFS and OS [35].

From a molecular perspective, recent advances in thyroid cancer research have highlighted multiple layers of biomarker development. Epigenetic dysregulation, such as DNA methylation, histone modifications, deregulation of microRNAs, and long non-coding RNAs, has been linked to tumor aggressiveness, RAI refractoriness, and *BRAF V600E* mutation. Immunohistochemical markers, including Ki-67, PD-L1 expression, and second-generation neuroendocrine markers, have also emerged as potential prognostic and predictive tools. Furthermore, liquid biopsy approaches, including circulating tumor DNA (ctDNA), plasma-derived exosomal miRNAs, and circulating long non-coding RNAs, offer a non-invasive means of monitoring tumor burden, resistance evolution, and potential therapeutic targets [36].

Although none of these molecular or circulating biomarkers have been prospectively validated, they provide biologically plausible links between host status, drug exposure, and clinical benefit. The integration of clinical indices, imaging markers, and molecular signatures may ultimately enable the development of a biomarker-driven personalized strategy to optimize LEN therapy for RAIR-DTC.

### 3.2. Neoadjuvant Use

#### 3.2.1. Clinical Experience and Real-World Applicability

In addition to its palliative role, LEN has demonstrated considerable potential as a neoadjuvant therapy in selected patients with locally advanced initially unresectable thyroid cancer. In cases involving extensive invasion into critical structures, such as the trachea, esophagus, or mediastinum, preoperative LEN may induce sufficient tumor shrinkage to permit surgical resection, a strategy known as conversion surgery. Achieving an R0 (clear surgical margin) or R1 (microscopically positive margin) resection status is particularly important in DTC, as it is strongly associated with improved prognosis [37].

An illustrative case involves a 66-year-old man with T4aN1aM1 papillary thyroid carcinoma presenting with airway obstruction, mediastinal invasion, and bilateral vocal cord paralysis, and was deemed unresectable. After an emergency tracheostomy, LEN was initiated at a dose of 24 mg/day. Two months later, imaging showed a 17% tumor reduction (RECIST v1.1) and regression of mediastinal invasion, enabling total thyroidectomy, total laryngectomy, and bilateral neck dissection via a cervical approach and avoiding more invasive mediastinal procedures. An R1 resection was achieved. Postoperatively, LEN was resumed at a reduced dose due to grade 3 proteinuria, hypertension, and tracheal hemorrhage; despite a dose reduction to 8 mg/day, disease control was maintained for several months. A *BRAFV600E* mutation was identified, and targeted therapy was reserved for future use. Subsequently, LEN was discontinued after the diagnosis of metastatic colorectal cancer was made. Importantly, local control of thyroid cancer achieved through LEN-based conversion surgery allowed the patient to smoothly transition to systemic therapy for colorectal cancer. At the final follow-up visit, the patient was alive with stable pulmonary metastases.

Neoadjuvant therapy is well established for several solid breast, esophageal, bladder, and lung cancers, for which preoperative systemic therapy facilitates tumor downstaging and increases the likelihood of curative resection [38,39,40,41,42,43,44]. However, the neoadjuvant approach is a relatively novel treatment for DTC but supported by accumulating case reports and small series. The reported tumor shrinkage rate is 20–70% (mean, 43.4%) over a treatment duration of 2 weeks to approximately 5 months, thereby enabling R0 or R1 resection in many cases. The mean preoperative drug withdrawal period was approximately 11.6 days, and serious wound-healing complications were rare (<0.5%), indicating favorable safety [45,46,47,48,49,50,51]. Although the optimal preoperative hold duration cannot be definitively standardized and should be tailored to individual patient and surgical factors, current evidence indicates that withholding LEN for approximately 10–14 days is a reasonable and commonly adopted approach. This practice helps minimize perioperative risks, provided that careful vigilance for wound healing and bleeding issues is maintained.

As summarized in Table 4, these findings demonstrate that neoadjuvant LEN can convert initially unresectable tumors, often due to invasion of the trachea, esophagus, or mediastinum, into surgically resectable tumors, thereby broadening subsequent therapeutic options, such as RAI and targeted agents. In selected cases, effective local control may reduce the systemic tumor burden, enabling the active treatment of coexisting malignancies.

However, in real-world clinical practice, the frequency of such cases remains relatively low, and most data are obtained from case reports or small retrospective cohorts. Although the outcomes are encouraging, they should be interpreted with caution. Prospective studies are required to define the true prevalence, safety, and long-term benefits of this approach.

#### 3.2.2. Evidence from Prospective Trials and Other Malignancies

Emerging evidence from other malignancies supports the use of this strategy. The LENS-HCC trial, a multicenter, single-arm, Phase II study of technically unresectable hepatocellular carcinoma, was completed and published [52]. Of the 49 included patients, 33 (67.3%) underwent surgery, and the conversion rates were particularly high in oncologically unresectable cases (76.2%). The 1-year OS rate was 75.9%, the median PFS was 7.2 months, and serious wound healing complications were rare. This trial provides strong prospective evidence that preoperative LEN can be administered safely, induces meaningful tumor shrinkage, and facilitates the surgical resection of locally advanced tumors. Prospective studies of thyroid cancer are currently underway. A Phase II trial of neoadjuvant LEN for invasive extrathyroidal DTC (NCT04321954) is actively recruiting patients to evaluate the impact of preoperative LEN on surgical outcomes and safety. In China, a single-arm trial of neoadjuvant LEN for locally advanced DTC was reported, with preliminary safety and efficacy data presented as an abstract in ESMO 2023 [53].

Other MKIs have been evaluated in this setting. A single-arm Phase II study of anlotinib (NCT04309136) in locally advanced DTC achieved an ORR of 76.9% and an R0/R1 resection rate of 61.5% in the intention-to-treat population (72.7% in the per-protocol population), with a median time to response of 61.5 days [54]. AEs such as hypertension and proteinuria were consistent with known toxicity profiles, and most were grade 1–2.

Immunotherapy-based neoadjuvant regimens show promise. A single-arm Phase II trial of toripalimab (anti-programmed death–1 antibody) combined with surufatinib (NCT04524884) reported an ORR of 60% and R0/R1 resection in nine of 10 patients, demonstrating a high rate of surgical conversion in this small cohort [55].

These prospective data strengthen the evidence base established by earlier case reports and small series, confirming the feasibility of preoperative molecularly targeted therapy-conversion surgery in thyroid cancer. Both single-agent MKIs (e.g., LEN, anlotinib, and surufatinib) and MKI–ICI combinations have achieved significant tumor shrinkage and resectability in initially unresectable disease.

As the results mature from the ongoing LEN trials (NCT04321954, ChiECRCT20210247) and Chinese neoadjuvant studies, these data will be critical in determining whether neoadjuvant LEN can be standardized as a core component of the multidisciplinary management of locally advanced thyroid cancer. If successful, neoadjuvant LEN could open the door to broader therapeutic strategies; securing local control through surgical resection enables safe RAI administration and allows the initiation of tailored systemic agents based on genetic profiling. Testing for actionable mutations such as *BRAFV600E* or *RET* can currently inform subsequent targeted therapy selection, thereby maximizing treatment personalization.

Accordingly, the NCCN Guidelines (v2.2024) recommend preoperative dabrafenib plus trametinib for resectable *BRAFV600E*-mutant anaplastic thyroid carcinoma (category 2B), reflecting broader acceptance of targeted neoadjuvant strategies for thyroid malignancies [8].

In summary, conversion surgery following neoadjuvant LEN is emerging as a transformative strategy for converting unresectable thyroid tumors to resectable tumors. By enabling surgical resection, RAI therapy, and personalized systemic treatment guided by genetic testing, this approach could significantly improve long-term outcomes in selected patients, particularly when integrated into multidisciplinary management pathways.

### 3.3. Combination with Immunotherapy

Building on the anti-angiogenic core mechanism summarized in Section 2.1, preclinical and translational studies have demonstrated that LEN can remodel the tumor microenvironment through vascular normalization and suppression of immunosuppressive cell populations. This dual mechanism provides a compelling biological rationale for combining LEN with ICIs.

Preclinical studies have shown that VEGFR blockade by LEN enhances CD8^+^ T-cell infiltration. It also reduces the activity of Tregs and myeloid-derived suppressor cells (MDSCs) and normalizes tumor vasculature. Multi-omics analyses further suggest that VEGFR/FGFR inhibition attenuates T-cell exhaustion and augments NK-cell cytotoxicity, thereby potentiating immune checkpoint blockade [56,57].

Early-phase clinical evidence supports this data. A U.S. multicenter phase II trial [58] demonstrated encouraging results with LEN plus pembrolizumab in patients with progressive RAIR-DTC. In MKI-naïve patients (cohort 1), the confirmed ORR was 65.5% with a median PFS of 26.8 months. In patients who progressed with LEN monotherapy (cohort 2), the ORR was 16%, and the median PFS was 10.0 months. Importantly, the AEs were consistent with the known profiles of each agent without any unexpected toxicities. In contrast, pembrolizumab monotherapy demonstrated limited activity against RAIR-DTC. In the KEYNOTE-028 trial (NCT02054806) [59], which enrolled 22 patients with advanced, PD-L1-positive thyroid cancer, the ORR was 9%, with only two partial responses. However, 59% of patients achieved stable disease, and the median PFS was approximately 7 months. Safety was generally manageable, with no unexpected immune-related adverse events. Taken together, these findings suggest that LEN plus pembrolizumab may substantially enhance the durability of responses compared with LEN monotherapy in LEN-naïve patients, while also providing a salvage option for those progressing after LEN. Moreover, contrast with pembrolizumab monotherapy highlights the potential importance of the anti-angiogenic and immune-modulating backbone provided by LEN in sensitizing thyroid tumors to immune checkpoint blockade.

Collectively, these findings establish LEN not only as a potent anti-angiogenic TKI but also as an immune-modulating backbone for rational combination strategies. Ongoing phase II/III studies will determine whether LEN–ICI regimens can achieve durable responses, overcome resistance to LEN monotherapy, and improve survival outcomes in patients with RAIR-DTC. These observations are consistent with a recent comprehensive review summarizing evolving combination strategies in RAIR-DTC, which highlighted LEN plus pembrolizumab and other TKI–ICI combinations (e.g., cabozantinib + atezolizumab) as promising investigational approaches to overcome resistance and improve durable responses [60].

### 3.4. Remaining Challenges

Despite significant advances achieved thus far, several critical issues remain unresolved and warrant systematic investigation. First, it is essential to establish standardized and proactive pathways for AE management that balance the need to maintain high-dose intensity in the early treatment phase with long-term tolerability. Second, the mechanisms underlying primary and acquired resistance must be elucidated to facilitate the development of rational co-targeting strategies. Third, the cost-effectiveness of prolonged therapy, particularly the incorporation of structured drug holidays to sustain RDI, requires careful evaluation. From a health-economic perspective, the high cost of long-term LEN therapy, including the expense of managing adverse events and hospitalization, is a major consideration. Proactive AE management and planned drug holidays, by reducing the incidence of severe events and potentially avoiding inpatient care, may lower overall healthcare expenditures and improve cost-effectiveness. Formal pharmacoeconomic analyses and real-world budget impact studies are required to quantify potential savings and guide reimbursement and policy decisions. Fourth, future clinical trials should prioritize the incorporation of patient-reported outcomes and quality of life as co-primary endpoints to ensure that therapeutic advances translate into meaningful patient benefits.

## 4. Limitations

The limitations of this review include the heterogeneity of patient backgrounds across studies and the absence of head-to-head comparisons between LEN and other systemic therapies. In particular, whether patients with RAIR-DTC at different clinical stages had varying response rates to LEN remains unclear, as most pivotal and real-world studies enrolled patients with progressive RAIR-DTC, irrespective of the baseline TNM stage. Moreover, many of the key conclusions presented in this review rely on post hoc analyses of the SELECT trial and retrospective real-world data and thus remain insufficiently validated in prospective clinical trials. Nevertheless, these exploratory and real-world findings provide valuable clinical insights that can assist in guiding daily decision-making and optimizing patient management. Future prospective studies stratified by clinical stage and designed to test these optimization strategies are required to clarify stage-specific efficacy and to confirm the hypotheses generated from retrospective and post hoc findings.

## 5. Conclusions

LEN’s emergence as a cornerstone therapy for RAIR-DTC is supported by compelling evidence from clinical trials and real-world practice. Its therapeutic potential is maximized through early initiation in appropriate patients, sustained dose intensity during the critical early phase, and the proactive management of AEs. Innovative strategies such as planned drug holidays, conversion surgery following neoadjuvant LEN, and third-line rechallenge or dose re-escalation offer additional avenues for extending disease control in selected populations. Nevertheless, challenges remain, including the need for optimized long-term AE management, strategies to overcome resistance mechanisms, and careful evaluation of cost-effectiveness and quality of life. The therapeutic landscape for advanced thyroid cancer continues to evolve. Therefore, LEN should be viewed not merely as a potent systemic agent but as a versatile platform for developing increasingly personalized, multidisciplinary treatment paradigms that not only preserve quality of life but also hold the potential to improve long-term survival.

## Figures and Tables

**Table 1:** Pivotal Clinical Trials and Key Real-World Evidence in Radioiodine-Refractory Differentiated Thyroid Cancer.

Trial (ClinicalTrials.gov No.)	Phase/Design	Enrolled Patients	Population	Intervention vs./Control	Primary Endpoint	Key Efficacy Outcomes	Key Grade ≥ 3 AEs
SELECT (NCT0132554)	Phase III, randomized, double-blind, placebo-controlled, multicenter	392 (LEN 261, PBO 131)	RAIR-DTC with radiographic progression ≤13 months; ECOG PS 0–2	Lenvatinib 24 mg QD vs. placebo	PFS (RECIST v1.1)	Median PFS: 18.3 vs. 3.6 months; HR 0.21 (95% CI 0.14–0.31, *p* < 0.001). ORR: 64.8% vs. 1.5%	Hypertension (44%), proteinuria (31%), diarrhea (8%), weight loss (10%), decreased appetite (5%)
DECISION (NCT00984282)	Phase III, randomized, double-blind, placebo-controlled, multicenter	417 (SOR 207, PBO 210)	Progressive RAIR-DTC; no prior TKI	Sorafenib 400 mg BID vs. placebo	PFS	Median PFS: 10.8 vs. 5.8 months; HR 0.59 (95% CI 0.45–0.76, *p* < 0.0001). ORR: 12.2% vs. 0.5%	Hand–foot skin reaction (20%), hypertension (9%), diarrhea (6%), hypocalcemia (4%)
COSMIC-311 (NCT03690388)	Phase III, randomized, double-blind, placebo-controlled, multicenter	258 (Cabo 170, PBO 88)	RAIR-DTC previously treated with VEGFR-targeted TKI (lenvatinib or sorafenib)	Cabozantinib 60 mg QD vs. placebo	PFS and ORR (blinded independent review)	Median PFS: 11.0 vs. 1.9 months; HR 0.22 (95% CI 0.15–0.32, *p* < 0.0001). ORR: 11.0% vs. 0%, *p* = 0.0003	Palmar–plantar erythrodysesthesia (10%), hypertension (9%), diarrhea (7%), fatigue (6%), hypocalcemia (3%)
Brose 2022 [10] (NCT02702388)	Phase II, randomized, open-label	152 (LEN 18 mg 76, LEN 24 mg 76)	RAIR-DTC, TKI-naïve	LEN 18 mg vs. 24 mg	ORR at 24 weeks and frequency of grade ≥3 TEAEs at 24 weeks	ORR 40.3%(18 mg) vs. 57.3%(24 mg); odds ratio (18/24) 0.50 (95% CI 0.26–0.96); Grade ≥ 3 TEAEs at 24 weeks: 57.1% vs. 61.3%;	Hypertension 15%(18 mg) vs. 19%(24 mg), proteinuria 4%(18 mg) vs. 5%(24 mg), and Asthenia 4%(18 mg) vs. 2%(24 mg)
Worden 2024 [7]	Real-world, retrospective, multicenter (U.S.)	308	RAIR-DTC, first-line Lenvatinib monotherapy	Lenvatinib (initial dose 24 mg QD in 62% of patients)	Real-world outcomes	rwBOR 72.4% (CR + PR); median rwPFS 49.0 months; OS rates: 78.4% at 24 months, 57.0% at 72 months; median OS not reached	Not reported

AE, adverse event; BID, twice daily; CI, confidence interval; ECOG PS, Eastern Cooperative Oncology Group performance status; HR, hazard ratio; ORR, objective response rate; PFS, progression-free survival; QD, once daily; RAIR-DTC, radioiodine-refractory differentiated thyroid cancer; RECIST, Response Evaluation Criteria in Solid Tumors; rwBOR, real-world best overall response; rwPFS, real-world progression-free survival; TKI, tyrosine kinase inhibitor; VEGFR, vascular endothelial growth factor receptor; TEAEs, treatment-emergent adverse events.

**Table 2:** Factors with significant differences between the lenvatinib and placebo arms.

Progression-Free		Overall Survival	
RAIR-DTC	HR, 0.20 (0.14–0.31) *p* < 0.001	≥65 years	HR, 0.53 (0.31–0.91) *p* = 0.020
Lung metastasis ≥10 mm	HR, 0.20 (0.15–0.27) *p* < 0.0001	Follicular thyroid cancer	HR, 0.36 (0.19–0.68) *p* = 0.002
		Lung metastasis ≥10 mm	HR, 0.63 (0.47–0.85) *p* = 0.025
		Baseline tumor burden ≥40 mm	HR, 0.42 (0.28–0.63) *p* = NE

HR, hazard ratio; NE, not estimated; RAIR-DTC, radioactive iodine-refractory differentiated thyroid cancer.

**Table 3:** Factors with significant differences within the lenvatinib arm.

Progression-Free Survival		Overall Survival	
Performance status 0 vs. 1	HR, 0.52 (0.35–0.77) *p* = 0.001	Performance status 0 vs. 1	HR, 0.42 (0.26–0.69) *p* = 0.0004
NLR ≤ 3 vs. >3	HR, 0.43 (0.29–0.65) *p* < 0.0001	NLR ≤ 3 vs. >3	HR, 0.48 (0.29–0.78) *p* = 0.0
		Baseline tumor burden ≤40 mm vs. >40 mm	HR, 0.42 (0.28–0.63) *p* = NE

HR, hazard ratio; NE, not estimable; NLR, neutrophil-to-lymphocyte ratio.

**Table 4:** Neoadjuvant therapy with lenvatinib for locally advanced thyroid cancer.

First Author	Age	Sex	Histology	TNM	Pretreatment Before LEN	Initial LEN Dose (mg)	Outcome	Treatment Duration	Off-Treatment Period	Surgery	Adjuvant Therapy
Iwasaki H	72	F	PTC	T4bN1bM1	None	20	30.8% reduction	4.7 mo	7 days	Yes	No
Iwasaki H	72	F	PTC	T4aN1bM0	None	14	20.0% reduction	3.1 mo	5 days	Yes	RAI
Iwasaki H	68	M	FTC	T4aN1bM1	None	24	33.3% reduction	2.9 mo	5 days	Yes	RAI
Iwasaki H	61	F	ATC	T4aN1bM1	None	24	42.0% reduction	2.0 mo	4 days	Yes	EBRT
Iwasaki H	66	F	PTC/ATC	T4aN1bM0	None	24	32.6% reduction	1.7 mo	10 days	Yes	No
Gay S	81	F	PDTC	T4aNXM0	EBRT 20Gy	10	Reduction, improvement in vocal function	8 w	2 w	Yes	No
Stewart KE	73	F	PTC	T4aN0M1	Sorafenib 800 mg × 4 w	24	31 × 59 × 32 mm → 17 × 28 × 22 mm	14 w	2 w	Yes	No
Tsuboi M	73	M	PTC	T4aN1bM0	None	14	Primary tumor 84% reduction, LN 45% reduction	22 w	17 days	Yes	RAI
Alshehri K	56	F	Not stated	—	EBRT 40Gy → Paclitaxel+Carboplatin → Doxorubicin → Sorafenib	10	90 × 90 × 44 mm → 72 × 66 × 37 mm	2.0 mo	4 w	Yes	No
Katoh H	65	F	PTC	T4bNXM0	None	24	40% reduction	2 w	10 mo	Yes	No
Golingan H	66	F	MTC	—	None	20	70% reduction	4 mo	3 days	Yes	No

Outcome–Lesion size (arrow (→) indicates reduction); ATC, anaplastic thyroid cancer; EBRT, external beam radiotherapy; FTC, follicular thyroid cancer.

## Data Availability

All data supporting the findings of this case series have been included in this article. Further inquiries can be directed to the corresponding author.

## Abbreviations

The following abbreviations are used in this manuscript:

AE	Adverse event
CI	Confidence interval
eGFR	Estimated glomerular filtration rate
HR	Hazard ratio
ICI	Immune checkpoint inhibitor
LEN	Lenvatinib
MKI	Multi-target tyrosine kinase inhibitor
NCCN	National Comprehensive Cancer Network
NE	Not estimable
NLR	Neutrophil-to-lymphocyte ratio
ORR	Objective response rate
OS	Overall survival
PBO	Placebo
PD	Progressive disease
PFS	Progression-free survival
PR	Partial response
PS	Performance status
RAI	Radioactive iodine
RAIR-DTC	Radioiodine-refractory differentiated thyroid cancer
RECIST v1.1	Response evaluation criteria in solid tumors version 1.1
TEAE	Treatment-emergent adverse event
TKI	Tyrosine kinase inhibitor
VEGF	Vascular endothelial growth factor
VEGFR	Vascular endothelial growth factor receptor
